# Significant change in the practice of chest radiography in Dutch ICUs

**DOI:** 10.1186/cc13440

**Published:** 2014-03-17

**Authors:** M Tolsma, TA Rijpstra, MJ Schultz, NJ Van der Meer

**Affiliations:** 1 University Medical Center Utrecht, the Netherlands; 2 Amphia Hospital, Breda, the Netherlands; 3 Academic Medical Center, Amsterdam, the Netherlands

## Introduction

>We performed a survey under Dutch intensivists on the current practice of chest radiography in their departments. Chest radiographs (CXRs) are obtained frequently in ICU patients, despite the diagnostic and therapeutic efficacy of routine CXRs being known to be low. The discussion regarding specific indications for CXRs in critically ill patients and the safety of abandoning routine CXRs is still continuing [1].

## Methods

A web-based questionnaire was sent to the medical director of all adult ICUs in the Netherlands. This survey contained questions regarding ICU characteristics, ICU patients, daily CXR strategies, indications for routine CXRs and the practice of radiologic evaluation.

## Results

Of the 83 ICUs that were contacted, 69 (83%) responded to the survey. Only 7% of ICUs still perform daily routine CXRs for all patients while 65% of ICUs say never to perform CXRs on a routine basis. A daily meeting with a radiologist is established in the majority of ICUs and is judged to be important or even essential. The therapeutic efficacy of routine CXRs was assumed by intensivists to be lower than 10% or to be between 10 and 20%. The efficacy of on-demand CXRs was assumed to be between 10 and 60%. There is consensus between intensivists to perform a routine CXR after endotrachial intubation, chest tube placement or central venous catheterization.

## Conclusion

The strategy of daily routine CXRs for critically ill patients has developed from a common practice (63%) in 2006 [2] to a rare practice (7%) nowadays. Intensivists still assume the value of routine CXRs to be higher than the efficacy that is reported in the literature. This might be due to the clinical value of negative findings, which has not been studied before.
